# Recurrence of Pelvic Chondrosarcoma through Fascial Defect into Abdominal Cavity

**DOI:** 10.1155/2014/674369

**Published:** 2014-11-12

**Authors:** Kemal Gökkuş, Tolgay Akin, Ergin Sagtas, Murat Saylik, Ahmet Turan Aydın

**Affiliations:** ^1^Private Memorial Antalya Hospital, Zafer Mahallesi, Yildirim Beyazit Caddesi, No. 91, Kepez, 07020 Antalya, Turkey; ^2^Orthopaedics and Trauma Department, Antalya Memorial Hospital, 07020 Antalya, Turkey; ^3^General Surgery Department, Antalya Memorial Hospital, 07020 Antalya, Turkey; ^4^Radiodiagnostic Department, Antalya Memorial Hospital, 07020 Antalya, Turkey; ^5^Orthopaedics and Trauma Department, Ozel Bahar Hospital, 16100 Bursa, Turkey

## Abstract

Our patient was a 76-year-old female who has been operated on 2 times in 8 years for pelvic chondrosarcoma. The patient came to our clinic with a large mass in left iliac region which extended into the paravertebral area. Physical examination and preoperative imagining studies revealed a mass at the left iliac area that infiltrated sciatic notch and extended from posterior iliac region towards the anterior side of iliac bone through the sciatic notch and an incisional hernia including descending colon. The mass was also penetrating the abdominal cavity through the hernia. Surgical intervention was planned. Since the tumor infiltrated the sciatic nerve, hemipelvectomy was indicated. Patient refused hemipelvectomy. Therefore, palliative debulking surgery was considered. We treated the case with marginal excision and abdominal wall reconstruction employing prolene and vicryl suture materials in order to prevent a postoperative visceral herniation and local invasion. At the latest follow-up appointment in 2 years, the patient still had no signs of tumor recurrence. This case showed us that an incisional hernia can serve as a pathway for the recurrence invasion of the chondrosarcoma.

## 1. Introduction

Chondrosarcomas constitute a heterogeneous group of neoplasms that have the production of cartilage matrix by tumor cells in common [1]. Chondrosarcoma is the third most common primary bone malignancy following myeloma and osteosarcoma, accounting for approximately 20% of bone sarcomas and mainly affecting the middle-aged population [2–4].

The pelvis is the most common site of occurrence with the ilium being the most frequently involved bone, followed by the pubis and ischium [5]. Although the majority of these tumors have a slow growth rate, rarely metastasize, and have a very good prognosis after surgery, local recurrence is a quite common occurrence, especially following inadequate excision. Since radiotherapy and chemotherapy were proven to be ineffective for the treatment of chondrosarcomas, wide en-bloc excision remains the treatment of choice [2, 6]. We hereby present a case of a second recurrent pelvic chondrosarcoma invading the pelvic region and locally extended into the abdominal cavity through the incisional hernia that caused by the abdominal wall defect which was a result of the previous operations. We treated the case with marginal excision and abdominal wall reconstruction employing prolene and vicryl suture materials in order to prevent a postoperative visceral herniation and local invasion.

## 2. Case

A 76-year-old woman came to our clinic with a large mass of the left iliac region.

According to the patient's history, she had a pelvic tumor removal surgery in 2006 and she was reoperated on for local recurrence in 2009. During admission to our oncosurgical clinic, an incisional hernia and a large mass of left iliac region were diagnosed. The patient was neurovascularly intact.

**Figure 1:**
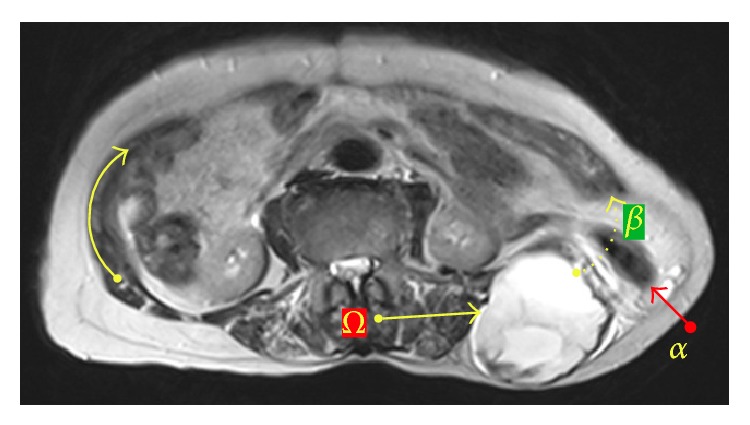
MRI imagining shows tumor (omega arrow), herniated descending colon (alpha arrow), and abdominal wall defect (dotted beta arrow).

**Figure 2:**
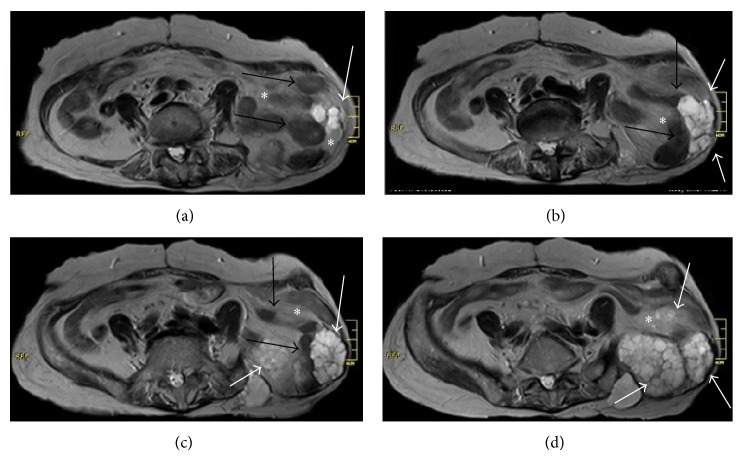
White arrows show tumoral mass (chondrosarcoma mass), black arrows show intestine or colon, and asterisk shows mesenteric fatty tissue.

Radiological studies showed a mass at the left iliac area that infiltrated sciatic notch and extended from posterior iliac region towards the anterior side of iliac bone through the sciatic notch and incisional hernia. The mass was also penetrating the abdominal cavity through the fascial defect and infiltrating the mesosigmoid that herniated through the fascial defect out of abdominal cavity (Figures 1 and 2). Surgical intervention was planned. Since the tumor infiltrated the sciatic nerve, hemipelvectomy was indicated. Patient refused hemipelvectomy. Therefore, palliative debulking surgery was considered.

The tumor was approached through the utilitarian pelvic incision that extended into the inner side of thigh and lateral border of the abdomen (the incision begins at the posterior inferior iliac spine and extends along the iliac crest to the anterior superior iliac spine. It is separated into two arms: one is carried along the inguinal ligament up to the symphysis pubis and the other turns distally over the anterior thigh for one-third the length of the thigh and then curves laterally just posterior to the shaft of the femur below the greater trochanter and follows the insertion of the gluteus maximus muscle).

**Figure 3:**
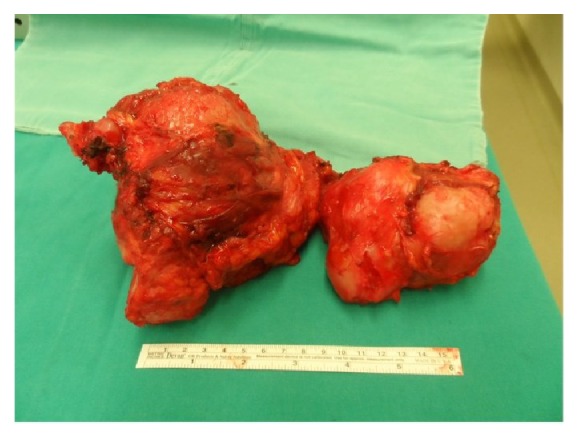
Excised tumor mass from the pelvic region.

**Figure 4:**
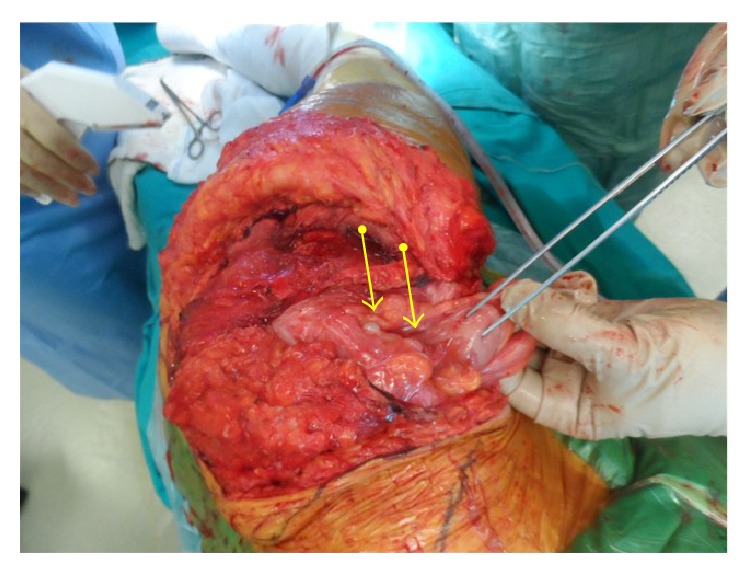
The intraoperative photograph, note the chondrosarcoma nodes in mesocolon (yellow arrows).

**Figure 5:**
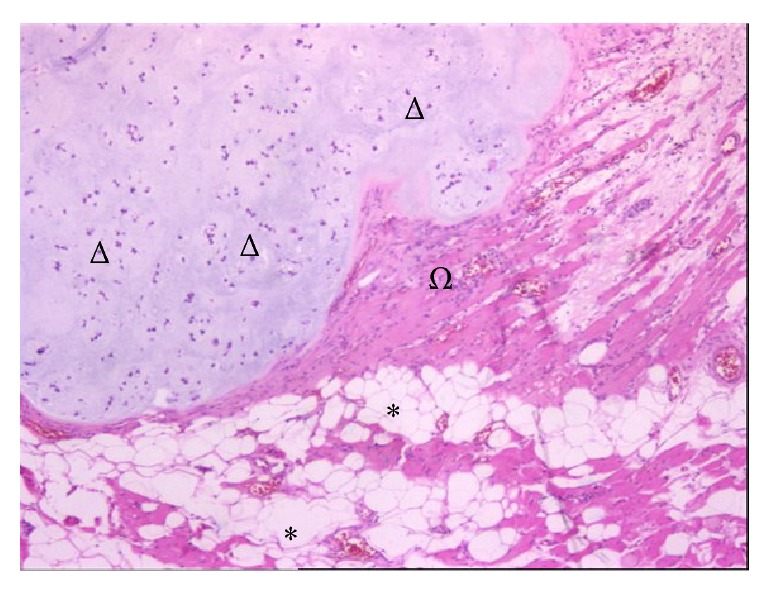
Hematoxylin-eosin stain with (x4) magnification. Asterisk: adipose tissue, omega: muscle tissue, and delta: atypical chondrocytes. Grade II chondrosarcoma.

The tumor in the pelvic and paravertebral area was marginally excised (Figure 3). Abdominal exploration was also performed using the same incision and tumors which infiltrated the omentum and mesosigmoid area were excised (Figure 4). Pelvic region and the abdominal cavity were separated using prolene and vicryl suture materials and hernia repair was done without using mesh. The operation lasted 8 hours. Recorded blood loss was 1500 mL and urine output was 1200 mL. The patient was transfused with 3 units of 500 mL erythrocyte suspension with sodium chloride-hydroxyethyl starch 120/0.4, 3000 mL Isolyte S, and 1 unit of fresh frozen plasma during the operation. In the post-op period, patient was held in Intensive Care Unit (ICU) for a day. During the ICU stay, 2 units of erythrocyte suspension and 1 unit of frozen plasma were used for transfusion. Two units of erythrocyte suspension were additionally transfused because of a drop in HB levels (8.2) on the 4th day of operation. Patient started mobilizing in bed on the 6th day and started to walk on the 9th day of the operation with assistance of a physiotherapist. Total hospital stay was 9 days. The patient was discharged without any complication. Patient had still no sign of tumor recurrence at the latest appointment of a 2-year follow-up period. Histological examination confirmed grade II chondrosarcoma diagnosis (Figure 5).

## 3. Discussion

Wide surgical tumor excision with adequate margins appears to be the procedure of choice in chondrosarcoma treatment, since this is the most effective way of reducing tumor recurrence rate [3–9]. Although local recurrence after inadequate surgical removal does not seem to significantly influence the overall survival, it must be kept in mind that each local recurrence bears the risk of the tumor evolving towards a higher-grade chondrosarcoma (up to 21.4%) [9].

The key point of our paper was to define an unusual way of local recurrence. To the best of our knowledge, no case of clearly defined pelvic chondrosarcoma developing local recurrence through the abdominal hernia into the abdomen was reported previously. Regarding the originality and rarity of our case, we performed a literature scan by employing PubMed database. The research criteria were as follows: (1) the research had to be in English, (2) papers examining pelvic chondrosarcoma related with colon and small intestines, (3) cases involving chondrosarcoma and abdominal wall repair, (4) cases involving bone resection that resulted abdominal hernia, and (5) cases involving chondrosarcoma, bone resection, and abdominal hernia altogether. Out of 252 articles, only 14 studies fulfilled the criteria described above. There was no case related with local recurrence and invasion into mesocolon area through the abdominal hernia. Considering this fact, we can say that our paper is unique in terms of focus on a local recurrence and invasion into mesocolon through the abdominal hernia.

Two articles (references [10, 11]) documented the involvement of small intestine and colon [10, 11].

Extraskeletal myxoid chondrosarcoma is differentiated by a biological structure that is distinct from the genetic heterogeneity observed in other forms of chondrosarcoma. Despite its high tendency to local recurrences and distant metastases, it has a relatively slow growth and a lazy clinical course [12, 13].

Bustinza-Linares et al. [10] presented a case of chest wall extraskeletal myxoid chondrosarcoma with small bowel metastasis (distant metastasis) which caused bowel obstruction. On the other hand, in our case the tumor locally invaded the omentum and sigmoid mesocolon through the abdominal wall defect and it might be described as local recurrence, as it is done in our case.

Hull [11] reported a case of chondrosarcoma originating from ribs (costochondral junction) that extended into colon. He performed a colon resection with wide resection of tumor together (resection of left 7th, 8th, 9th, 10th, 11th, and 12th ribs with a portion sternum with splenic flexura of the colon). He repaired the abdominal wall using tantalum mesh to prevent visceral hernia. No recurrence was reported during the 9-month follow-up period. This paper is important because of the emphasis on both colon involvement and abdominal wall repair (this article was similar to our case regarding abdominal involvement and abdominal wall repair. Even in this article, the link between the abdominal incisional hernia and local recurrence or invasion of chondrosarcoma through hernia has not been shown).

We searched the literature for the link between bone resection (the resected bone was used for bone grafting) and abdominal hernia (cases involving bone resection that resulted with abdominal hernia) and 7 articles were found [14–20].

Petsatodis et al. performed radical excision of the tumor (chondrosarcoma) at symphysis pubis and used synthetic mesh in order to reduce the possibility of a postoperative visceral herniation [21]. They emphasized the importance of abdominal wall repair. No recurrence was reported during the 2-year follow-up period.

Juan-García et al. [22] reported abdominal hernia after resection of a chondrosarcoma of the pelvis. In contrast with our case, their case had no local recurrence invasion through the hernia. The link between the abdominal incisional hernia and local recurrence or invasion of chondrosarcoma through hernia was not discussed. We would like to stress the contribution to literature by presenting the evidence about this relationship.

In our case we repaired abdominal wall carefully with prolene and vicryl suture materials to prevent future herniation and local invasion of pelvic chondrosarcoma.

This case showed us that an incisional hernia can serve as a pathway for the recurrence invasion of the chondrosarcoma.

In conclusion, surgeons should separate compartments by materials like mesh or sutures to minimize the ratio of intercompartmental recurrence.
